# Rewiring the stomatal lineage: A novel developmental pathway to plant totipotency

**DOI:** 10.1111/jipb.70067

**Published:** 2025-10-31

**Authors:** Kang Chong

**Affiliations:** ^1^ State Key Laboratory of Forage Breeding‐by‐Design and Utilization Institute of Botany, the Chinese Academy of Sciences Beijing 100093 China; ^2^ University, of Chinese Academy of Sciences Beijing 100049 China; ^3^ China National Botanical Garden Beijing 100093 China

## Abstract

This commentary highlights a study revealing that stomatal lineage precursor cells can be reprogrammed into somatic embryos through a LEC2–SPCH–YUC auxin regulatory circuit. This study uncovers a developmental route to plant totipotency and offers promising strategies for improving regeneration efficiency in difficult‐to‐transform crop species.

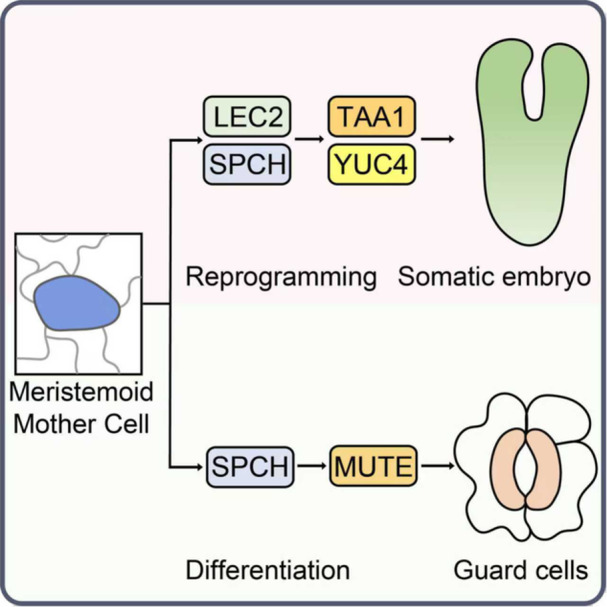

Plant tissue culture is a powerful and widely used tool for clonal propagation in modern agriculture and forestry, as well as in plant transgenic engineering. Successful tissue culture depends on a dynamic trade‐off between hormone levels, such as auxin and cytokinin, and the specific localization of reactive oxygen species on a given genetic background. In the tissue culture process, one of the major bottlenecks is the precise control of somatic cell differentiation into embryos. As is known, somatic embryogenesis (SE) is the process by which somatic cells are reprogrammed to form embryos, representing one of the most remarkable demonstrations of plant cellular plasticity. Since its discovery in carrot cultures in the 1950s (Steward et al., 1958; Reinert, 1959), SE has been widely used for clonal propagation and genetic transformation, as well as being a powerful model to study plant regeneration and totipotency. However, the underlying mechanisms remain poorly understood. For example, less is known about how embryos can originate directly from somatic cells without an intervening callus phase. A long‐standing question is which somatic cell types can serve as the origin of plant totipotency, especially in species or tissues that are recalcitrant to regeneration. In a recent study published in *Cell*, Tang et al. (2025) identified a specific somatic cell type as the origin of totipotency, providing key insight into this fundamental process.

Classical SE protocols rely on external hormone application or stress treatments, obscuring the endogenous triggers that permit reprogramming. Pioneering work has identified key regulators, such as *LEC1*, *LEC2*, *BBM*, and *WUS* (Boutilier et al., 2002; Lotan et al., 1998; Stone et al., 2001; Zuo et al., 2002), yet how these factors interact with developmental pathways and hormone dynamics to induce totipotency *in vivo* has not been resolved fully. Recent studies have begun to bridge this gap. In rice, the zygotic transcription factor BBM1 activates early embryonic programs via YUCCA‐mediated auxin biosynthesis (Khanday et al., 2023). In *Arabidopsis*, genome‐wide chromatin accessibility analyses have indicated that auxin rapidly remodels the chromatin to promote the progressive acquisition of embryogenic identity (Wang et al., 2020). Still, a lineage‐resolved map of SE initiation in intact tissues, without exogenous chemical induction or wounding, has been lacking.

In a recent *Cell* study, Tang et al. (2025) tackled this question by defining a direct route to totipotency from a specific, well defined epidermal cell type. Using high‐resolution imaging, lineage tracing, and single‐cell transcriptomics, they showed that the precursors of the stomatal lineage cells, the meristemoid mother cells (MMCs), could directly initiate SE without an intervening callus phase. This discovery clarifies the cellular origin of plant totipotency in this context and uncovers a developmental bifurcation between normal stomatal development and embryogenic reprogramming (Figure 1).

**Figure 1:**
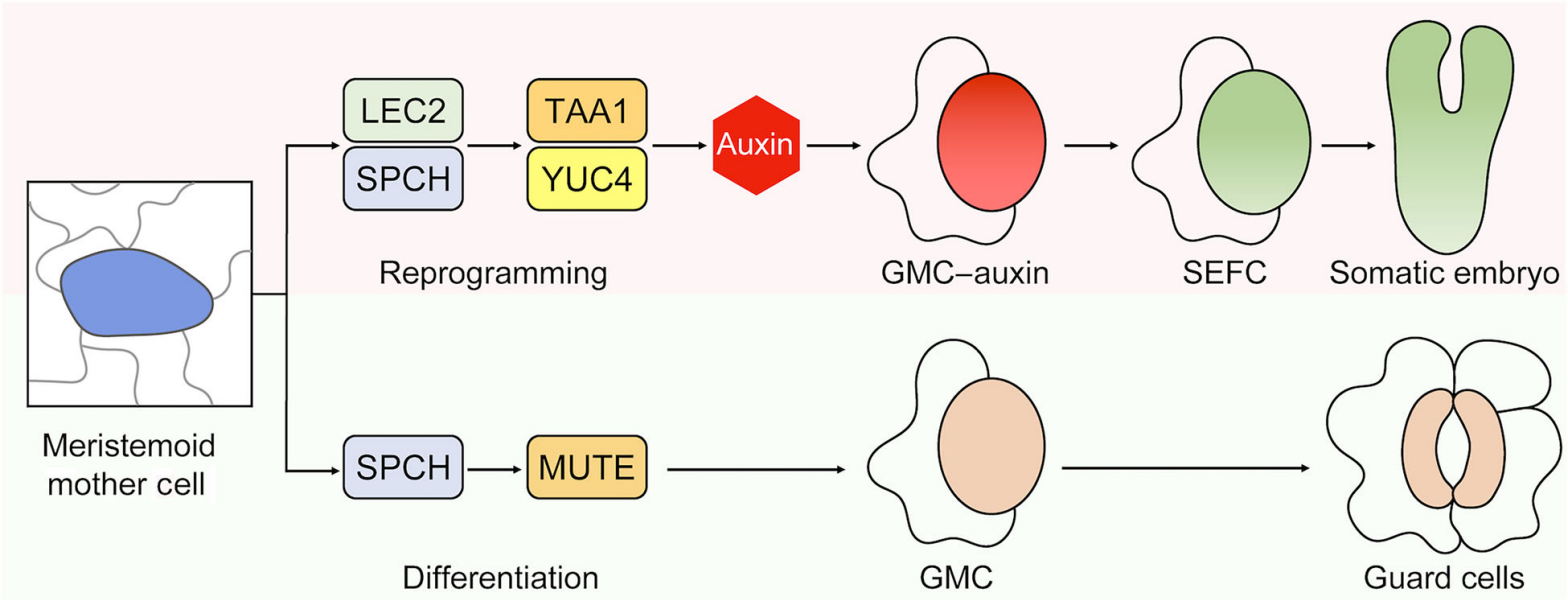
A novel developmental route from stomatal lineage to somatic embryogenesis Meristemoid mother cells (MMCs) specified by SPEECHLESS (SPCH) can be reprogrammed by LEAFY COTYLEDON2 (LEC2) into somatic embryo founder cells (SEFCs). LEC2 and SPCH cooperatively activate TAA1 and YUC4, triggering localized auxin biosynthesis. This establishes a transient GMC–auxin intermediate state that diverts cells from the stomatal lineage toward totipotent reprogramming, enabling somatic embryos to arise directly from single epidermal cells without an intervening callus phase.

Mechanistically, Tang et al. identified a transient intermediate, termed the guard mother cell (GMC)–auxin state, that arose from MMCs as they diverged from the stomatal lineage. The GMC–auxin state was marked by a localized rise in auxin biosynthesis and signaling, together with increased activity of chromatin regulators and translational machinery. Cells entering the GMC–auxin state either proceeded toward guard mother cell fate and complete stomatal differentiation, or transitioned into somatic embryo founder cells (SEFCs), which seed embryogenesis. Central to this fate switch is the embryogenesis regulator LEAFY COTYLEDON2 (LEC2). LEC2 physically and functionally cooperates with the stomatal regulator SPEECHLESS (SPCH) to directly activate auxin biosynthetic genes *TAA1* and *YUC4*, thereby establishing a local auxin circuit that promotes SEFC identity in a cell‑autonomous manner. Pharmacological and genetic evidence indicate that endogenous, localized auxin biosynthesis is indispensable for this reprogramming and cannot be replaced by exogenous auxin analogs such as 2,4‑D. These results establish localized auxin biosynthesis as both necessary and sufficient for this cell fate transition (Sakamoto et al., 2022).

To capture the dynamics of this transition, Tang et al. used single‐nucleus RNA sequencing of more than 69,000 nuclei to map the developmental trajectory from MMCs toward either guard cells or SEFCs. This analysis revealed a transient GMC–auxin cluster at the bifurcation point, marked by co‐expression of late stomatal and early embryogenic regulators such as SERK1 and ABI3. Genes linked to auxin transport, signaling, and developmental pathways, such as *ERECTA* and *MP/ARF5*, were strongly upregulated, highlighting a complex network that integrated hormonal and developmental cues to govern the transition to totipotency (Figure 1).

Beyond its mechanistic insight, this study provides a conceptual breakthrough by demonstrating that plant totipotency can emerge directly from a single, specified cell type *in vivo*, rather than from a dedifferentiated mass of cells. The discovery that MMCs, which are normally committed to stomatal development, harbor latent embryogenic potential also suggests that other lineages may contain comparable gateways to regeneration. Practically, the LEC2–SPCH–YUC circuit provides a blueprint for rationally engineering regenerative competence in economically important, but regeneration resistant, crops. Instead of applying broad hormone treatments, researchers could now target specific lineages with a tailored combination of transcription factors and spatially restricted signals, potentially overcoming genotype‐dependent barriers. Similar strategies have been explored in cereals, in which BBM1‐driven YUC activation triggers zygote‐like programs in the scutellum (Khanday et al., 2023).

While transformative, the study leaves several open questions. The data from experiments that relied on induced *LEC2* overexpression in *Arabidopsis* seedlings may not fully reflect natural reprogramming events. Whether similar pathways operate under physiological conditions or in other lineages remains to be tested. Furthermore, the identification of chromatin regulators such as BPC1 suggests a multi‐layered control system. Future work using single‐cell epigenomics tools could clarify how transcriptional and epigenetic regulation interact to enable reprogramming. Finally, it will be important to test whether analogous lineage‑specific entry points to totipotency exist across species, and to define the minimal combinations of factors and localized auxin circuits required to access them. Combining single‑cell multi‑omics, advanced imaging, and targeted perturbations, as well as extending these approaches to regeneration‑recalcitrant crops, could transform this conceptual framework into practical strategies for engineering plant totipotency.

Nevertheless, this study provides a roadmap for translating these fundamental discoveries into next‐generation tools for crop biotechnology. Building on the concept of lineage‐specific reprogramming, synthetic biology could be used to design minimal, switchable reprogramming circuits that transiently activate auxin biosynthesis in targeted cells. Advances in tissue culture engineering, such as microfluidic systems and spatially patterned hormone delivery, may recreate the endogenous signaling environments needed to stabilize totipotent states. Together, these approaches may establish a technological foundation for systematically engineering plant regeneration across diverse species.

In summary, Tang et al. have delivered a landmark study that redefines the cellular and molecular origin of SE. By identifying the GMC–auxin intermediate as a pivotal node, they have shown how localized auxin biosynthesis and transcriptional regulators rewire developmental trajectories toward SE. This study reframes regeneration as a precise developmental alternative embedded within normal growth. It offers a roadmap for engineering regeneration in recalcitrant crops through targeted, lineage‐specific strategies, rather than broad hormone treatments.

## CONFLICTS OF INTEREST

The author declares no conflicts of interest.

## AUTHOR CONTRIBUTIONS

K.C. conceptualized, wrote and revised the manuscript.
